# Spatiotemporal neural network dynamics for the processing of dynamic facial expressions

**DOI:** 10.1038/srep12432

**Published:** 2015-07-24

**Authors:** Wataru Sato, Takanori Kochiyama, Shota Uono

**Affiliations:** 1 Department of Neurodevelopmental Psychiatry, Habilitation and Rehabilitation, Graduate School of Medicine, Kyoto University, 53 Shogoin-Kawaharacho, Sakyo, Kyoto 606-8507, Japan; 2 Brain Activity Imaging Center, Advanced Telecommunications Research Institute International, 2-2-2 Hikaridai, Seika-cho, Soraku-gun, Kyoto 619-0288, Japan

## Abstract

The dynamic facial expressions of emotion automatically elicit multifaceted psychological activities; however, the temporal profiles and dynamic interaction patterns of brain activities remain unknown. We investigated these issues using magnetoencephalography. Participants passively observed dynamic facial expressions of fear and happiness, or dynamic mosaics. Source-reconstruction analyses utilizing functional magnetic-resonance imaging data revealed higher activation in broad regions of the bilateral occipital and temporal cortices in response to dynamic facial expressions than in response to dynamic mosaics at 150–200 ms and some later time points. The right inferior frontal gyrus exhibited higher activity for dynamic faces versus mosaics at 300–350 ms. Dynamic causal-modeling analyses revealed that dynamic faces activated the dual visual routes and visual–motor route. Superior influences of feedforward and feedback connections were identified before and after 200 ms, respectively. These results indicate that hierarchical, bidirectional neural network dynamics within a few hundred milliseconds implement the processing of dynamic facial expressions.

Dynamic changes in facial expressions of emotion are a particularly valuable source of information in face-to-face interactions. From an evolutionary perspective^1^, the human mind has evolved to process the dynamic facial expressions of conspecifics efficiently. Behavioral studies have revealed that dynamic facial expressions automatically induce multiple psychological activities such as perceptual enhancement^2^, emotional responses^3^, and facial mimicry^4^.

Consistent with these behavioral data, neuroimaging studies using functional magnetic resonance imaging (fMRI) and positron-emission tomography have shown that several cortical and subcortical regions are more active when viewing dynamic facial expressions compared to control conditions^5^,^6^,^7^,^8^,^9^,^10^. The cortical regions consistently include the human V5 region, fusiform gyrus (FG), superior temporal sulcus (STS), and inferior frontal gyrus (IFG).

However, the temporal profile of the activity in these brain regions in response to dynamic facial expressions remains unclear. To understand the neural mechanisms, that is, the causal relationships among the brain regions, temporal information is indispensable^11^. Electrophysiological studies involving recording electroencephalography (EEG), magnetoencephalography (MEG), or intracranial EEG are more appropriate for understanding this issue at a higher temporal resolution. However, the few relevant electrophysiological studies have reported inconsistent findings^12^,^13^,^14^,^15^,^16^,^17^,^18^. For example, an EEG study found that dynamic faces, compared to dynamic mosaics, activated the posterior cortices at about 170 ms, and the researchers speculated that the source of these activities would be within the STS^12^. This contrasts with an MEG study, which reported that the current dipole of posterior activity for dynamic facial stimuli during this time period was located in the V5 region^13^. These inconsistencies are mainly caused by limitations in the spatial resolution of the electrophysiological measures^19^.

Furthermore, no studies have empirically tested the neural network dynamics (i.e., dynamic causal relationships among the brain regions) involved in the processing of dynamic facial expressions at a millisecond temporal resolution. It has been theoretically postulated that multiple brain regions would constitute the functional network involved in processing dynamic faces^20^,^21^, and some previous neuroimaging studies have attempted to reveal these neural-interaction patterns by analyzing hemodynamic responses (e.g., Ref. 22). However, electrical neuronal communication is implemented rapidly within only a few hundred milliseconds^23^, and thus electrophysiological data analysis would be more appropriate for depicting such rapid networking patterns.

Here we recorded MEG signals while participants viewed dynamic facial expressions of fear and happiness as well as dynamic mosaics. We presented computer-morphed dynamic facial expressions, which were shown to activate the widespread brain regions in previous neuroimaging studies^24^. To investigate the automatic processes in response to dynamic facial expressions, participants passively observed stimuli with dummy tasks. To depict brain activities with high spatial and temporal resolutions, we conducted source-reconstruction analysis utilizing fMRI data^25^. To investigate the neural network dynamics over distributed brain regions that process dynamic facial expressions, we further conducted dynamic causal modeling (DCM)^26^.

## Results

### Behavioral performance

Performance on the dummy target-detection task was perfect and sufficiently rapid (correct identification rate = 100.0%; mean ± *SD* reaction time = 420.0 ± 64.4 ms).

### Regional brain activity

MEG data (Supplementary Fig. 1) were subjected to fMRI-constrained MEG source reconstruction^24^, and then analyzed using the random-effects general linear model (GLM) including stimulus type (dynamic facial expression versus dynamic mosaic), emotion (fear versus happiness), and time window (0–50, 50–100, 100–150, 150–200, 200–250, 250–300, 300–350, and 350–400 ms) as repeated-measures factors of interest.

**Figure 1:**
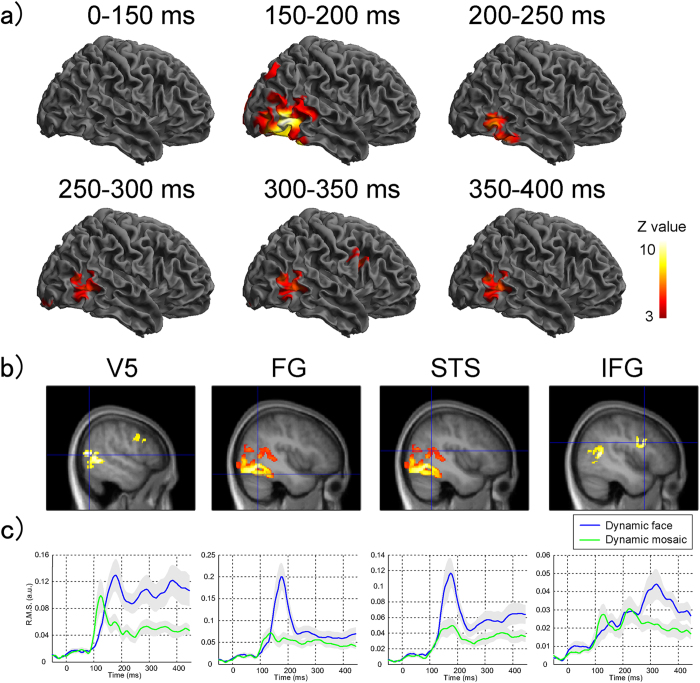
Regional brain activity analysis. (**a**) Statistical parametric maps (SPMs) showing brain regions activated in response to dynamic facial expressions versus dynamic mosaics at each 50-ms time window during 0–400 ms after the stimulus onset. The areas of activation are rendered on spatially-normalized brains. Left and right columns depict activities in the left and right hemispheres, respectively. The extent threshold of p < .05 corrected for multiple comparisons with a height threshold of p < .01 (uncorrected). (b,c) SPMs (**b**) and waveforms of source estimates (**c**) in response to dynamic facial expressions versus dynamic mosaics in the regions of interest. The SPMs are overlaid on the normalized anatomical magnetic resonance image of one of the participants. The extent and height thresholds are identical to those in the above mentioned maps.

The main effect of stimulus type, which contrasted dynamic facial expressions and dynamic mosaics (Table 1; Fig. 1), did not reveal any significant activation during the time windows of 0–50, 50–100, or 100–150 ms. Broad ranges of bilateral posterior regions were significantly activated during 150–200 ms, including the activation foci of the middle temporal gyrus adjacent to the inferior temporal sulcus, corresponding to the human V5^27^, FG, and STS. Although the activation patterns changed slightly, significant activation of the posterior cortices was observed across all of the later time windows. In addition, at 300–350 ms, significant activation was observed in the right IFG.

The main effect of emotion and the interaction between the stimulus type and emotion were also tested in each time window; however, no brain region showed significant activation.

Figure 1c shows the averaged root mean square (RMS) time course of source activities in the regions of interest (ROIs). Prominent peaks of activity in the V5, FG, and STS were observed about 170 ms after the stimulus onset, clearly discriminating the dynamic facial expressions and mosaics. These could also be differentiated in the same regions after 200 ms with visible small peaks. Although there were small peaks of IFG activity during 100–200 ms and 200–300 ms, there were no differences between stimulus type conditions. The peak at about 320 ms differentiated the dynamic facial expressions and mosaics in the IFG.

### DCM

**Figure 2:**
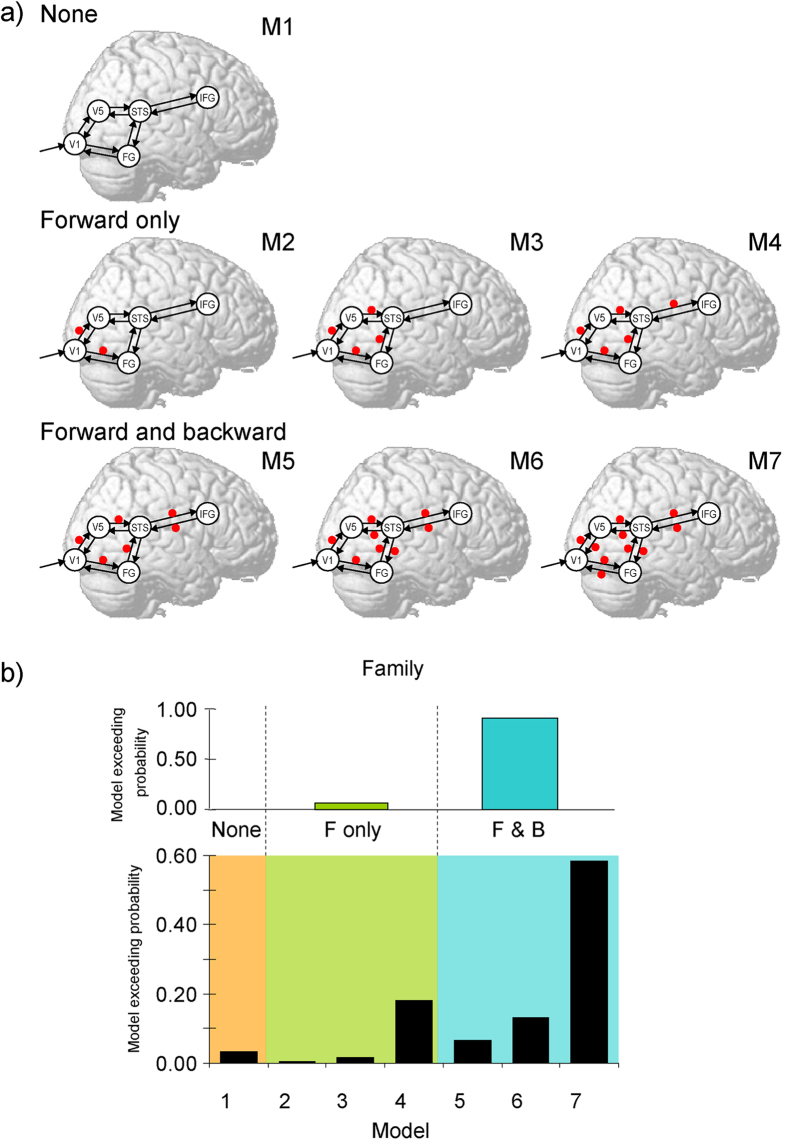
Models and results of dynamic causal modeling. (**a**) Analyzed model. Arrows indicate intrinsic connections between brain regions. Red points indicate the possible locations of the modulatory effect of dynamic facial expression. Candidates included the models with modulatory effects on no connection (upper), only forward connections (middle), and both forward and backward connections (lower). (**b**) Exceedance probabilities of models (upper) and model families (lower: none, forward [F] alone, forward and backward [F&B] in Bayesian model selection).

DCM analyses were conducted to test our models (Fig. 2a). To define the interacting cortical network, we adopted a combination of the dual visual routes model^20^ and the visual–motor route model^21^ to process dynamic social signals. For the dual visual routes model, Oram and Perrett^20^ suggested that cortical visual processing involves the ventral and dorsal pathways that send outputs to the STS region, which acts as a convergence zone. This model is supported by several physiological^28^ and anatomical^29^ studies in monkeys. For the visual–motor route model, de Antonia and Hamilton^21^ proposed that the direct functional connectivity between the STS and IFG implements motor mimicry. Consistent with this, several anatomical studies in humans^30^ and nonhuman primates^31^ have shown that the STS and IFG are directly connected. Based on these findings, we hypothesized that the cortical network, in which the dorsal (i.e., V1–V5) and ventral (i.e., V1–FG) pathways converge on the STS, which interacts with the IFG, is involved in the processing of dynamic facial expressions. We examined whether these connections could be modulated during the processing of dynamic facial expressions and whether connectivity modulation was observed only in the forward connections or in both the forward and backward connections. Several computational theories have pointed out that such differences could have a significant impact on cognitive functions^32^,^33^.

Random-effects Bayesian model selection (BMS) was applied with different locations of modulatory effects for dynamic facial expression. The BMS exceedance probability was highest for the model including modulatory effects in all forward and backward connections (Fig. 2b). Comparisons of model families confirmed that models with modulatory effects on both forward and backward connections better accounted for the data than did models without modulation or those with only forward-connection modulation.

**Figure 3:**
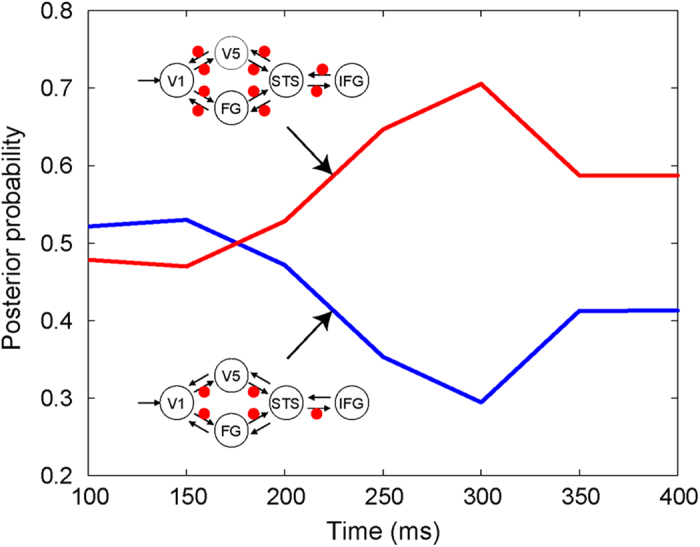
Results of increasing time-window dynamic causal modeling. Posterior probabilities in the Bayesian model selection of each time window (ranging from 100 to 400 ms in 50-ms increments) for models without (blue) and with (red) modulatory effects on backward connections.

To further elucidate the neural coupling temporal profile, specifically the importance of feedback modulation, we compared models with and without modulatory effects on backward connections over post-stimulus time windows of 100–400 ms in 50-ms increments. The random-effects BMS showed that, although the model without backward-connection modulation fitted better with the data until 150 ms, the model with backward modulation better accounted for the data after 200 ms (Fig. 3).

## Discussion

### Spatiotemporal profile of brain activity

Our regional brain activity results showed that observation of dynamic facial expressions, compared with dynamic mosaics, activated distributed cortical regions, including the V5, FG, STS, and IFG. The activation of these regions is consistent with the findings of previous neuroimaging studies^7^. However, because neuroimaging techniques measure neuronal activity using only indirect hemodynamic responses, our results extend these findings, indicating that the electrical activity of these regions is enhanced during the observation of dynamic facial expressions. The activation of these brain regions in response to dynamic facial expressions is consistent with the results of previous EEG or MEG recordings and their source localizations that reported the activities of the V5^13^, FG^16^,^18^, STS^16^, and IFG^18^. However, ours is the first study to depict the activities of all of these widespread brain regions. We believe that our MEG recording^34^ and/or fMRI-constrained source reconstruction analysis^24^ improves the spatial resolution of those electrophysiological recordings.

More importantly, our results depict a time course of regional brain activity in response to dynamic facial expressions. Higher activity was observed as multiple peaks within 400 ms of the stimulus onset. Because dynamic facial expressions are ecologically valid stimuli, our results suggest that perception of facial expressions during daily social interactions rapidly activates a widely distributed neural network. Information regarding the temporal profile of regional brain activities allows a more complete understanding of the cognitive functions of the brain regions and causal relationships among them for the processing of facial expressions.

At 150–200 ms, peaking around 170 ms, broad ranges of posterior regions were activated, including the V5, FG, and STS. This result is consistent with previous electrophysiological studies^12^,^13^,^14^,^15^,^16^,^17^, although none identified the whole of these regions. The functions of these regions were previously suggested to be motion analysis^5^, invariant feature analysis^7^, and the integration of form and motion^12^ for dynamic facial expressions, respectively. Our results suggest that these various types of visual analysis of dynamic facial expressions are implemented at this early time stage.

These posterior regions also showed some activation for dynamic facial expressions after 200 ms. This is consistent with a previous electrophysiological study^16^, suggesting that these regions are involved in multiple processes associated with dynamic facial expressions. Neuroimaging studies have revealed activation of these regions in several psychological tasks. For example, the FG was active both in face perception and the personal identification of facial stimuli^35^, whereas the STS was active in the presentation of dynamic facial stimuli as well as the evaluation of participant facial intention^36^. Behavioral studies showed that dynamic facial expressions elicit various cognitive activities such as subjective perception^2^ and emotion recognition^37^. Together with these studies, our results suggest that the posterior regions related to visual analysis of dynamic facial expressions at 150–200 ms are also involved in the cognitive evaluation of faces after 200 ms.

At 300–350 ms, the IFG showed heightened activation in response to dynamic facial expressions. The relatively late activity in the IFG is consistent with the result of previous EEG studies, which reported that dynamic facial expressions elicited evident frontal region activities at 200–350 ms^17^ and large source activities in the IFG at 200–300 ms^18^. Our finding is also in line with a previous EEG study demonstrating that the IFG was active at 332–400 ms while viewing dynamic hand actions^38^. These data suggest that IFG activation around 300 ms is involved in the processing of the actions of others, not specific to effectors. Previous neuroimaging studies reported that this region is not only activated when participants passively observe dynamic facial expressions^25^, but also when they simultaneously imitate facial expressions^39^. This is consistent with the theory that the IFG contains mirror neurons that match the observation and execution of facial expressions^40^. Consistent with this, previous behavioral studies reported that the observation of dynamic facial expressions induced congruent facial muscle activity at around 500–1,000 ms from stimulus onset^4^. Together with these data, our results suggest that mirrored motor activation for dynamic facial expressions is implemented by IFG activity at about 300 ms.

These results indicate that neural activation in response to dynamic facial expressions change depending on the time stage: the visual and motor-related cortices are active in turn. These data suggest that the cognitive representations of facial expressions may also qualitatively change over time. Debate remains whether motor representations are necessary for facial expression recognition^41^ or not^42^. Our data suggest that the brain manipulates visual/cognitive representations first, and then utilizes motor representations for the processing of dynamic facial expressions around 300 ms. Notably, however, our effective connectivity analyses (discussed below) revealed that feedback information from the IFG started to affect the visual cortices at about 200 ms. Before accomplishing the motor resonance, preliminary analysis of motor representations might be utilized for computations in the visual areas during the processing of dynamic facial expressions.

We did not observe any main effects or interactions for emotion. Consistent with this, neuroimaging studies previously found comparable activity in several cortical regions in response to dynamic facial expressions of positive and negative emotions^7^. Our results extended these findings, indicating that the timing of cortical activation is also comparable for the processing of such facial expressions.

### Neural network dynamics

Our DCM results showed that the observation of dynamic facial expressions modulated functional interactions rapidly among the brain regions. The best fitting model included modulation of both dorsal and ventral pathways and the pathway from the STS to the IFG. These pathways have been theoretically proposed to be involved in the processing of dynamic social signals^20^,^21^ and were speculated to be involved in facial expression processing^43^. However, to the best of our knowledge, our study provides the first empirical evidence in humans showing that these neural pathways are involved in processing dynamic facial expressions.

Our results revealed that the observation of dynamic facial expressions modulates forward and backward connections in the neural network. These results are consistent with several theoretical proposals that information is transmitted bidirectionally in the neural computation of social interactions^32^,^33^, although the details remain unclear. For example, Iacoboni^32^ proposed that during the observation of the dynamic social signals of others, the visual representations of observed actions are first converted into ones’ own motor plans through forward projections, and then the motor plans provide predictive information to refine visual-representation processing via backward projections. Kilner *et al*.^33^ proposed that this type of computation during action observation could be implemented by minimizing prediction error through forward and backward connections between cortical areas. Some behavioral studies corroborate this perspective of feedback modulation from motor to perceptual/cognitive processing for dynamic facial expressions. For example, mimicking the manipulations of participants’ faces facilitated emotion recognition of dynamic facial expressions^44^.

Our analyses further specified the time regions in which forward and backward connections were activated during facial-expression processing. In response to dynamic facial expressions, the brain initially activated whole systems in a feedforward manner until 150 ms, and then utilized both feedforward and feedback activation after 200 ms. Interestingly, several electrophysiological studies have reported that brain activity after 200 ms is related to the conscious perception of visual stimuli^45^. Recording and stimulation studies also showed that re-entrant activities of the visual cortices via feedback projections are related to the production of conscious awareness for visual stimuli^46^. These data suggest that the facilitation of re-entrant neural activation after 200 ms while observing dynamic facial expressions satisfies the conditions for enhancing subjective perceptions. Consistent with this idea, a behavioral study showed that the conscious perception of facial expressions is facilitated by dynamic presentations^2^. Taken together, our data might depict spatiotemporal neural dynamics changing from unconscious to conscious processing of dynamic facial expressions.

### Implications and limitations

Our approach could be applicable to divergent lines of research. One application is the investigation of psychiatric disorders involving social impairments, such as autism spectrum disorders (ASD). ASD individuals are characterized primarily by deficient communication via emotional facial expressions^47^; however, the underlying neural mechanism remains controversial. A previous neuroimaging study revealed that ASD individuals show hypoactivation in some social brain regions while viewing dynamic facial expressions, including the V5 and STS^48^. For future research, it would be interesting to investigate the spatiotemporal neural dynamics of their impaired dynamic facial-expression processing.

The present study had some limitations. First, we asked participants to engage in a dummy task to ensure that brain activity could be considered as primarily reflecting automatic processes for dynamic facial expressions. However, this task did not reveal the details of cognitive functions associated with brain activities, and different tasks might enhance or suppress the activity of certain brain regions at particular time points. For example, a previous neuroimaging study reported heightened STS activity during the intentional recognition of emotional faces^49^; however, its temporal profile remains unknown. In future studies, participants should be asked to engage in intentional cognitive processes in response to dynamic facial expressions to specify the functions of brain activity.

Second, we used dynamic mosaic images to control for low-level visual features such as dynamic information. Because the dynamic mosaics lacked the information found in faces, the relationship of the spatiotemporal components described in our study to the processing of dynamic and static facial expressions is not clear. In a previous fMRI study, we compared brain activity elicited by dynamic mosaics and static facial expressions and found similar spatial patterns^7^. However, the present data do not allow us to draw conclusions about commonality in the temporal patterns of dynamic and static stimuli. The comparison between dynamic and static facial expressions is an important topic for future research.

Third, a dynamic neutral expression condition was not tested. Because of this limitation, whether the current findings could be specific to dynamic emotional expressions remains unclear. It was difficult to generate dynamic face stimuli with dynamic properties comparable with those of dynamic emotional expressions and neutralize all emotional meaning; emotional facial expressions contain complex motions involving multiple facial parts^50^, and even facial motions not included in prototypical emotional expressions can transmit emotional messages^51^. Possible options for investigation of this issue may include backward presentations of emotional facial expressions as presented in some neuroimaging studies^52^,^53^. Future research comparing emotional versus neutral dynamic faces would be necessary to better understand the neural mechanism involved in the processing of dynamic facial expressions.

Fourth, stimuli were presented only in the center of the visual field. This was because we aimed to investigate the temporal dynamics of brain activities for centrally presented dynamic facial expressions reported in a previous neuroimaging study^7^. However, it is known that the central presentation of stimuli induces a cancellation effect of electric activities in the early visual areas in MEG recordings^54^. Hence, null results in the early visual areas may be attributable to this factor. Future research investigating stimulus presentation in the peripheral visual field^55^ may be promising to further investigate the early stages of neural processing of dynamic facial expressions.

Fifth, only a single speed of dynamic facial expressions was tested. Although the speed we used closely reflected natural changes in dynamic facial expressions^25^, and hence the current results could be applicable to explain neural activities in daily life, whether dynamic facial expressions of different speeds may elicit brain activation at different time stages remains unclear. This would be an interesting matter for future research.

Finally, our analyses were restricted to the event-related potential (ERP) model. Although this method is conventional and valid, some recent electrophysiological studies have suggested that the ERP may not detect rapid activities in high frequency bands^56^. Consistent with this notion, some previous studies using brain stimulation^57^ and recording somatosensory EEG^58^ reported that visual or tactile brain activity involved in face processing may occur rapidly, even before 100 ms. It may be possible that our analysis could not detect these rapid components because of the limitations of the analysis due to temporal resolution. Future studies applying other data analysis methods, such as time-frequency analysis, may extend the understanding of spatiotemporal neural dynamics for processing dynamic facial expressions.

In summary, our MEG recording depicted the temporal profiles of rapid activation patterns in some cortical regions, including the V5, FG, STS, and IFG, in response to dynamic facial expressions versus dynamic mosaic images. Our analysis further revealed that these brain regions exhibited hierarchical and bidirectional dynamic interactions. These results elucidate the spatiotemporal neural network dynamics involved in the processing of dynamic facial expressions.

## Methods

### Participants

Fifteen volunteers (six females and nine males; mean ± *SD* age, 26.9 ± 3.9 years) participated in the study. All participants were right-handed and had normal or corrected-to-normal visual acuity. All of the participants were Japanese. Each participant gave written informed consent after the procedure was fully explained. This study was approved by the ethics committee of the Primate Research Institute, Kyoto University. The study was also conducted in accord with the Declaration of Helsinki.

### Experimental design

The experiment involved a within-participant two-factorial design, with stimulus type (dynamic facial expression versus dynamic mosaic) and emotion (fear versus happiness).

### Stimuli

The raw materials were grayscale photographs of the faces of eight individuals chosen from a standard set^59^ depicting fearful, happy, and neutral expressions. None of the faces were familiar to any of the participants.

For the dynamic expression stimuli, computer animated clips of emotional facial expressions were generated from the photographs. Initially, 24 intermediate images between neutral (0%) and emotional (100%) expressions were created in 4% steps using morphing software (FUTON System, ATR) implemented on a computer running Linux. To create a moving clip, 25 images from 4%–100% were presented in succession. This morphing stimuli set was used in several previous behavioral studies, and was shown to elicit appropriate behavioral responses, including perceptual enhancement^2^, spontaneous facial mimicry^4^, and subjective emotional reactions^60^. The set was also used in previous neuroimaging studies, and was shown to elicit widespread neural activation^7^. Each image was presented for 20 ms, and thus each animation clip lasted for 500 ms. A previous behavioral study confirmed that this speed was recognized as closely reflecting natural changes that occur in the dynamic facial expressions of fear and happiness^25^. The stimuli subtended a visual angle of 15.0° vertical × 10.0° horizontal.

The dynamic mosaics were made from the same materials. The abovementioned facial images were divided into 18 vertical × 12 horizontal (0.8° vertical × 0.8° horizontal of visual angle) squares and were randomly reordered using a constant algorithm. Next, a set of 25 images corresponding to the original dynamic expression images was serially presented as a moving clip. The presentation speed was identical to that of the dynamic expressions. These manipulations resulted in similar levels of size, brightness, and dynamic information for both the dynamic mosaic images and the corresponding original dynamic-expression stimuli. These dynamic mosaics were used in several previous behavioral (e.g., ref. 60) and neuroimaging (e.g., ref. 7) studies and revealed clear differences from dynamic facial expressions.

### Presentation apparatus

The events were controlled by Presentation software version 10.0 (Neurobehavioral System). The stimuli were projected from a liquid crystal projector (DLA-G150CL, Victor) to a mirror positioned in front of the participants.

### Procedure

The experiment was conducted in an electromagnetically shielded room. Each stimulus was presented seven times. In addition, a red cross was presented as the target in 28 trials, yielding a total of 252 trials for each participant. Stimuli were presented in a random order. In each non-target trial, the stimulus was presented centrally for 500 ms following the appearance of a cross for 500 ms at a fixation point. In each target trial, participants were asked to detect the red cross and press a button with the right forefinger as quickly as possible. These dummy tasks confirmed that the participants were attentive and prevented the explicit processing of the stimuli content. Post-hoc debriefing confirmed that the participants were unaware that the purpose of the experiment involved the investigation of faces. Participants were also instructed not to blink while stimuli were presented. The inter-trial interval varied from 1,800 to 2,400 ms. To avoid habituation and drowsiness, participants were given short rests upon completion of 36 trials. Before data collection, participants were familiarized with the procedure through training involving a block of 14 trials.

### MEG acquisition

MEG data were obtained in an electromagnetically shielded room using a 210-channel, whole-head supine-position system (PQ1400RM; Yokogawa). A forehead strap was used to stabilize the head position. MEG data were sampled at 1,000 Hz through a band-pass of 0.05–200 Hz. Vertical and horizontal electrooculograms (EOGs) were recorded simultaneously.

To measure the head position within the MEG-sensor system, five head-position indicator coils were mounted on the participants’ heads. Electromagnetic calibration of the coil positions was conducted before each MEG recording session. The participant’s head shape and calibration coil positions were digitized using a three-dimensional laser-optical scanner and a stylus marker (FastSCAN Cobra, Polhemus), and were used later to coregister the MEG sensor locations to an anatomical space defined by each individual MRI.

### Anatomical MRI acquisition

This was performed on a 3T scanning system (MAGNETOM Trio, A Tim System; Siemens) using a 12-channel head coil. A T1-weighted high-resolution anatomical image was obtained using a magnetization prepared rapid-acquisition gradient-echo (MP-RAGE) sequence (TR = 2,250 ms; TE = 3.06 ms; IT = 900 ms; flip angle = 9°; field of view = 256 × 256 mm; voxel size = 1 × 1 × 1 mm).

### Data analysis

#### fMRI prior

The data analyses were performed using SPM8 r4290 (http://www.fil.ion.ucl.ac.uk/spm) implemented in MATLAB R2009a (Mathworks).

To make an empirical prior on MEG source reconstruction^24^, we re-analyzed the fMRI data from a previous study^7^. In this study, the stimuli were made from the same materials as those in the present study. After the standard preprocessing and group statistics^61^, we created a statistical parametric map of *T*-statistics (SPM{*T*}) for the comparison of dynamic facial expression versus dynamic mosaic, and then thresholded at p < .001 for the height threshold and k >100 voxels (800 mm^3^) for the extent threshold. The resulting activations showed similar patterns to the original study, including the clusters of bilateral posterior visual areas (the activation foci in the V5, FG, and STS), the right inferior parietal lobule, and the right IFG. We adopted these cortical activities as the prior.

#### MEG preprocessing

Continuous MEG data were epoched into 500-ms segments for each trial and down-sampled to 200 Hz; pre-stimulus baseline data were collected for 50 ms, and experimental data were collected for 450 ms after the stimulus onset. The data were initially subjected to independent component analyses for the purpose of artifact rejection using EEGLAB toolbox (http://sccn.ucsd.edu/eeglab/index.html). Threshold-based artifact rejection was also conducted. Any epochs containing a gradiometer amplitude of ≥3,000 fT/cm and an EOG amplitude of ≥80 μV were rejected as artifacts. Trials including artifacts were also excluded on the basis of visual inspection. The frequencies of artifact-contaminated trials did not differ across conditions (mean ± *SD* 10.4 ± 3.8%; p = .97, within-participant analysis of variance). The pre-processed data were then low-pass filtered at 48 Hz, baseline corrected on the basis of the 50-ms pre-stimulus period, and averaged over trials by conditions for subsequent analyses.

Before the source reconstruction analysis, we conducted a sensor level analysis to check for data quality by computing the mean-square field strength from the MEG sensors. The mean-squared responses were then averaged across all participants to create the grand-mean waveforms for each condition and contour maps at representative peaks (Supplementary Fig. 1).

For fMRI-constrained MEG source reconstruction^24^, an anatomical MRI of each participant was segmented and spatially normalized to the Montreal Neurological Institute (MNI) space. The inverse of this normalization transformation was then used to warp a canonical cortical mesh in the MNI space to the individual cortical mesh^62^. The cortical mesh described the source locations with 20,484 vertices (i.e., “fine” size). Next, the MEG sensors were coregistered to the anatomical MRI by matching the positions of three fiducials (nasion and R- and L-preauricular points) and head shape. The forward model could then be computed using a “single shell” model^63^ by assuming that the orientations of the sources were normal to the cortical mesh.

Following inversion of the forward model, we conducted cortical source reconstruction using a parametric empirical Bayesian framework^62^. A standard minimum norm inversion was used to compute the cortical source activities on the cortical mesh based on the aforementioned fMRI data as spatial priors on the source localization^24^. The use of priors in the current framework imposed only soft (not hard) constraints^64^. The parameters of the inversion were based on SPM default settings, with the exception of not using a Hanning taper for the time series.

For each participant and condition, we obtained three-dimensional source-reconstructed images in the MNI space of the averaged evoked activity every 50 ms between 0–400 ms in the post-stimulus window. The intensity was normalized to the mean over voxels and conditions to reduce inter-participant variance. Finally, these source reconstructed images were smoothed with an 8 mm full-width at half-maximum isotropic Gaussian kernel to improve the signal-to-noise ratio and to compensate for anatomical variability among participants.

#### MEG regional brain activity analysis

MEG source-reconstructed images were entered into the random-effects GLM including stimulus type (dynamic facial expression versus dynamic mosaic), emotion (fear versus happiness), and time window (0–50, 50–100, 100–150, 150–200, 200–250, 250–300, 300–350, and 350–400 ms) as repeated-measures factors of interest; participant was a factor of no interest. A non-sphericity correction was used to correct for uneven variance between the factor levels. The observations dependent on the factor levels were also corrected. The ensuing covariance components were estimated using a restricted maximum likelihood procedure and used to adjust the statistics. The low-variance regions, which can cause artificially high statistical values and localization bias, were also adjusted^65^.

Planned contrasts were performed for each time window. We tested the main effect of stimulus type (dynamic facial expression versus dynamic mosaic) and also analyzed the main effect of emotion and the interactions between stimulus type and emotion for descriptive purposes. Statistical inferences were performed using SPM{*T*} based on the random field theory^66^. Significantly activated clusters were identified if they reached the extent threshold of *p* < .05 corrected for multiple comparisons across the entire brain, with a height threshold of *p* < .001 (uncorrected).

To display activation waveforms, the RMS time course of MEG source activity within a 4-mm radius of the peak focus was extracted between 0–450 ms for each participant, and then averaged across participants.

#### DCM

We used DCM for ERP modeling of electrophysiological data^67^ to explore how effective connectivity between brain regions was modulated by dynamic facial expression. DCM allows us to make inferences about the influence that one neural system exerts over another and how this is affected by experimental contexts^26^. We focused on modulation of the cortical network by the presentation of dynamic facial expressions; thus, individual averaged responses were collapsed across the frightened and happy conditions, and the factor of emotion was excluded from the DCM input.

Based on our hypothesis, we selected the following five ROIs in the right hemisphere: the V1 (x 22, y − 84, z − 4), V5 (x 54, y − 64, z 8), FG (x 42, y − 60, z − 10), STS (x 42, y − 52, z 14), and IFG (x 44, y 12, z 26). The coordinates of the latter four regions were defined based on the results of the main effect of stimulus type (dynamic facial expression versus dynamic mosaic) at 300–350, 150–200, 150–200, and 300–350 ms, respectively. Anatomical identification was conducted using the cytoarchitectonic map with the Anatomy Toolbox version 1.5^27^. The V1 coordinate was derived from the strongest activation focus in response to all stimuli presentations compared to the baseline at 100–150 ms. The time window was determined because it was the first to show a large deflection during visual inspections of source estimates in this region^68^. The V1 search region was derived from the Anatomy Toolbox. The ROIs were restricted to the right hemisphere because this was the only one that showed significant activation in all ROIs.

Each source was modeled by a single equivalent current dipole method with prior fixed locations and a variance of 4 mm. The hypothesized models of neural networks were constructed with the driving input of the visual stimulus into V1. The prior of onset time (the inversion algorithm optimized this parameter^31^) was set at 80 ms based on the values reported in previous DCM studies (e.g., 96 ms^67^) and visual inspection of the current data. The bidirectional (forward and backward) intrinsic connections were constructed for V1–V5, V1–FG, V5–STS, FG–STS, and STS–IFG. The modulatory effect of dynamic facial expressions was modeled to modulate each of these bidirectional connections. We assumed that the model included two paths (forward and backward) at three stages of V1–V5/FG, V5/FG–STS, and STS–IFG. Based on these criteria, we constructed a total of seven models by changing the locations of the modulatory effects (Fig. 2a). The first model included no modulatory effect on any connections. The next three models included modulatory effects on forward connections, but differed in terms of the included stages. The last three models included modulation on backward connections, in addition to modulatory effects on all forward connections, and also differed gradually in terms of the included stages. To select the fittest model, we used random-effects BMS^69^. We used the exceedance probability to evaluate the belief that a particular model was more likely than any other given the group data.

To clarify the involvement of feedback modulation, we grouped the models into three families: no modulation family, only including the null modulation model; forward modulation only family, including modulatory effects on forward connections alone; and forward and backward modulation family, containing modulatory effects on both forward and backward connections. We then compared the families using BMS^70^.

To specify the effect of timing of backward modulation, we further compared the models with and without backward modulation (models 4 and 7, respectively, in Fig. 2a) using an increasing time-window approach^23^. Random-effects BMS was performed on eight data segments, with lengths increasing from 100 to 400 ms in 50-ms increments after the stimulus onset.

## Additional Information

**How to cite this article**: Sato, W. *et al*. Spatiotemporal neural network dynamics for the processing of dynamic facial expressions. *Sci. Rep*. **5**, 12432; doi: 10.1038/srep12432 (2015).

## Supplementary Material

Supplementary Information

## Figures and Tables

**Table 1:** Brain regions showing significant activation in response to dynamic facial expressions versus dynamic mosaics.

Time (ms)	Anatomical region	MNI coordinate			Z-value	Cluster size (mm^3^)
		X	Y	Z		
0–50	None					
50–100	None					
100–150	None					
150–200	R inferior temporal gyrus	50	–62	–6	8.33	33912
	R inferior temporal gyrus	54	–66	–2	8.23	
	R fusiform gyrus	42	–60	–20	7.75	
	R inferior occipital gyrus	42	–78	–2	7.36	
	R middle temporal gyrus	48	–46	18	5.51	
	R middle temporal gyrus	42	–52	14	4.25	
	L inferior occipital gyrus	–48	–76	–12	8.03	31968
	L fusiform gyrus	–38	–58	–20	7.82	
	L middle occipital gyrus	–40	–70	6	7.12	
	L inferior temporal gyrus	–58	–56	–6	6.09	
	L middle temporal gyrus	–60	–50	2	4.79	
	L middle occipital gyrus	–16	–100	10	6.11	10184
	L calcarine sulcus	–4	–94	6	5.23	
200–250	R inferior temporal gyrus	56	–62	–8	5.87	9504
	R fusiform gyrus	42	–40	–24	4.1	
	L inferior temporal gyrus	–52	–54	–12	4.24	2936
250–300	R middle temporal gyrus	54	–56	0	4.8	6848
	R middle temporal gyrus	54	–66	10	4.71	
	R lingual gyrus	12	–86	–8	4.1	4704
	R calcarine sulcus	8	–80	2	4.09	
300–350	L middle occipital gyrus	–46	–74	12	5.13	10232
	L inferior occipital gyrus	–50	–80	–2	3.74	
	R middle temporal gyrus	54	–64	8	5.27	7320
	R middle temporal gyrus	46	–52	8	4.91	
	R calcarine sulcus	8	–80	2	4.38	4472
	R inferior frontal gyrus	44	12	26	4.71	2560
350–400	L middle temporal gyrus	–46	–74	12	5.69	13056
	L inferior occipital gyrus	–48	–74	–4	4.75	
	R middle occipital gyrus	54	–64	8	5.38	7216
	R middle temporal gyrus	46	–54	8	5.21	
	L inferior temporal gyrus	–64	–46	–12	3.93	2680
