# Supplementary material for: Spatiotemporal neural network dynamics for the processing of dynamic facial expressions
Source: Sci Rep. 2015 Jul 24;5:12432. doi: 10.1038/srep12432 (PMC4513292; doi:10.1038/srep12432)
Supplement: Supplementary Information [file srep12432-s1.pdf]

Spatiotemporal neural network dynamics for the processing of dynamic facial expressions

Wataru Sato, Takanori Kochiyama, and Shota Uono

Supplementary data

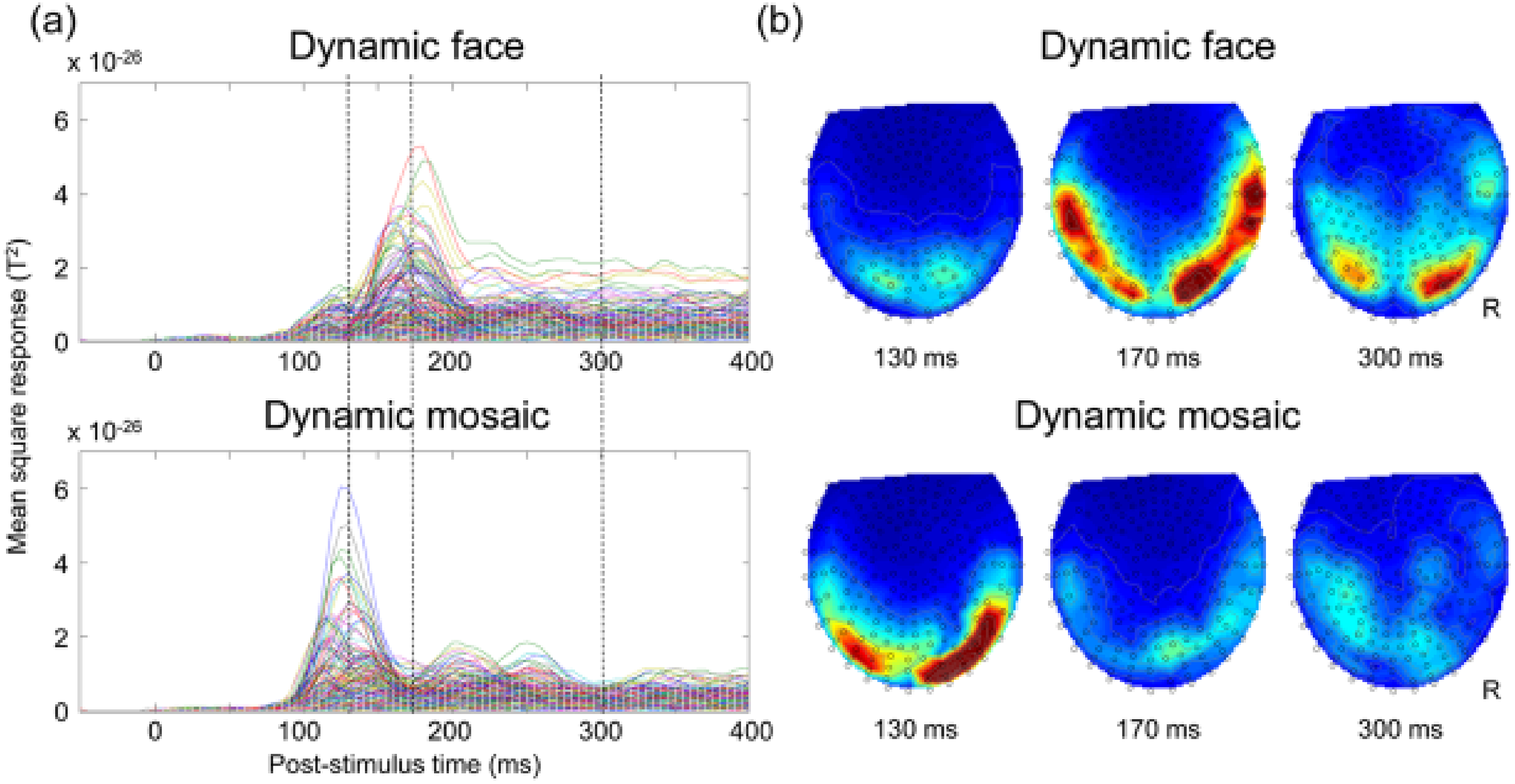

Supplementary Figure 1. Magnetoencephalography data at sensor level.

(a) Mean-square responses averaged across all participants on a sensor-by-sensor basis in response to dynamic facial expressions versus dynamic mosaics.

(b) Grand-averaged field contour maps in response to dynamic facial expressions (upper row) versus dynamic mosaics at representative peaks (lower row). R = right.
